# Modulation of the major histocompatibility complex by neural stem cell-derived neurotrophic factors used for regenerative therapy in a rat model of stroke

**DOI:** 10.1186/1479-5876-8-77

**Published:** 2010-08-20

**Authors:** Chongran Sun, Han Zhang, Jin Li, Hua Huang, Hongbin Cheng, Yajie Wang, Ping Li, Yihua An

**Affiliations:** 1 Department of Neural Stem Cell, Beijing Neurosurgical Institute, Beijing Tiantan Hospital, Capital Medical University, China; 2 Department of Neurosurgery, 2nd Affiliated Hospital of Zhejiang University Medical College, Hangzhou, China; 3 Department of Laboratory, Beijing Tiantan Hospital, Capital Medical University, Beijing, China; 4 Department of Electrophysiology, Beijing Tiantan Hospital, Capital Medical University, Beijing, China

## Abstract

**Background:**

The relationship between functional improvements in ischemic rats given a neural stem cell (NSC) transplant and the modulation of the class I major histocompatibility complex (MHC) mediated by NSC-derived neurotrophins was investigated.

**Methods:**

The levels of gene expression of nerve growth factor (NGF), brain-derived neurotropic factor (BDNF) and neurotrophin-3 (NT-3) were assayed from cultures of cortical NSC from Sprague-Dawley rat E16 embryos. The levels of translated NGF in spent culture media from NSC cultures and the cerebral spinal fluid (CSF) of rats with and without NGF injection or NSC transplant were also measured.

**Results:**

We found a significant increase of *NGF*, *BDNF *and *NT-3 *transcripts and NGF proteins in both the NSC cultures and the CSF of the rats. The immunochemical staining for MHC in brain sections and the enzyme-linked immunosorbent assay of CSF were carried out in sham-operated rats and rats with surgically induced focal cerebral ischemia. These groups were further divided into animals that did and did not receive NGF administration or NSC transplant into the cisterna magna. Our results show an up-regulation of class I MHC in the ischemic rats with NGF and NSC administration. The extent of caspase-III immunoreactivity was comparable among three arms in the ischemic rats.

**Conclusion:**

Readouts of somatosensory evoked potential and the trap channel test illustrated improvements in the neurological function of ischemic rats treated with NGF administration and NSC transplant.

## Introduction

Ischemic stroke is a common neurological disorder and is one of the leading causes of casualty worldwide. It is caused by the occlusion of a cerebral artery with thrombi and emboli, which leads to an infarction and the death of neural tissue. Current treatments are primarily palliative and are useful to only a minority of patients after stroke. Currently, there is no effective treatment for restoring the neurological functions lost during a stroke. Recent studies in pre-clinical and clinical trials have shown that stem cell-based therapy can lead to symptomatic relief and may offer a novel potential treatment [1]. Nevertheless, the underlying therapeutic mechanisms for neural repair and the induction of functional improvement remains controversial.

The ability of neural stem cells (NSC) to differentiate into neural cells has been seen in culture [2]. Given the complexity of both the structure and function of the central nervous system (CNS), it is critical to understand the mechanisms by which transplanted neural cells can replace the damaged cells and interact with healthy host cells in a well-organized manner. Cell-based therapy might elicit a chaperone effect in the at-risk neural tissue surrounding the lesioned area *via *the up-regulation of neurotrophic and neuroprotective factors, which help to promote the survival, migration and differentiation of endogenous precursors after stroke [3].

In rats, the administration of nerve growth factor (NGF) has been shown to enhance the expression of the class I major histocompatibility complex (MHC) in neurons, but not in glial cells, and decrease the expression of the class II MHC in glias [4]. Immune response and inflammation are common sources of secondary injury in neural cells after stroke. *In vitro *cultures have been used to demonstrate that NSC, neurons and glias express both class I and II MHCs, which were recently recognized to be crucial in the activity-dependent refinement and plasticity of neural connections in the developing and adult CNS [5]. We hypothesized that the functional improvements in ischemic rats given NSC transplant might be related to modulation of the class I MHC, mediated by NSC-derived neurotrophins in the lesioned micro-environment of the CNS.

## Materials and methods

### Culture of Neural stem cells

Neural stem cells were harvested from the cortex of E16 Sprague-Dawley rat embryos. The head was decapitated and the whole brain was removed from the skull. Meninges, choroid plexus and coherent blood vessels were carefully stripped off. The tissue was cut into small pieces, triturated with a glass pipette and allowed to pass through a 28-mesh copper sieve to remove large chunks. After three washes with Dulbecco's modified Eagle's medium (DMEM; Sigma-Aldrich, St. Louis, http://www.sigmaaldrich.com), 1.5×10^7 ^cells were seeded in 15 mL of high-glucose DMEM/F12 (Sigma-Aldrich, St. Louis) supplemented with 2% B27 (Gibco, Carlsbad, CA, http://www.invitrogen.com), 20 μg/L basic fibroblast growth factor (FGF, PeproTech, NJ, http://www.peprotech.com) and 20 μg/L epidermal growth factor (EGF, PeproTech, NJ) onto a 75 cm^2 ^non-adherent tissue culture flask (Laixin, Shanghai, China, http://www.lx17.cebiz.cn) and maintained at 37°C in a humidified environment with 5% CO_2_. Cultures were passaged with 0.25% tripsin and titration with a glass pipette once a week, and half of the spent culture media was replaced.

### Enzyme-linked immunosorbent assay (ELISA) of NGF

One-day-old medium from the first seven passages of NSC cultures and 80 μL of cerebral spinal fluid (CSF) from 40 Sprague-Dawley rats were collected and centrifuged at 400 g for 10 min to remove cellular debris. The supernatant was stored at -80°C. An ELISA kit (Boster, Hubei, China, http://www.boster.com.cn) was utilized, following the manufacturer's protocol, to quantify the NGF present in the culture supernatants and CSF. Briefly, 100 μL of sample and standards were added to plates that were pre-coated with monoclonal anti-NGF and allowed to react for 1.5 h at 37°C. Samples were washed thoroughly, and then incubated with 100 μL of biotin-conjugated anti-NGF at 37°C for 1 h. Plates were washed to remove the unbound anti-NGF and incubated again with 100 μL of streptavidin-conjugated horseradish peroxidase at 37°C for 30 min. Signals were developed by adding 100 μL of chromogenic tetramethylbenzidine. The reaction was arrested after 15 min with 100 μL of stopping solution. The absorbance was read at 450 nm. The color intensity of this reaction is proportional to the amount of bound NGF. A standardization plot was established for NGF standards at 250, 125, 62.5, 31.3, 15.6, 7.8 and 3.9 pg/mL. The diluting buffer was used as the negative control. All measurements were performed in triplicate.

### Molecular Analyses

#### RNA extraction and cDNA transcription

To test the total RNA, positive and negative controls were extracted using the RNAqueous^®^-Midi Kit and the manufacturer's protocol (Applied Biosystems, Foster City, CA, http://www.ambion.com). Briefly, cells were disrupted with the lysis buffer composed of a high concentration of guanidinium salt. The lysate was diluted with a 64% ethanol solution and passed through the glass fiber filter in the RNAqueous Filter Cartridge. RNA bound to the filter while other cellular contents flowed through it. The Filter Cartridge was washed with the wash solutions to remove contaminants, and the RNA was then eluted using the Elution Solution of very low ionic strength. RNA integrity was monitored for two sharp intense bands, 18s and 28s, by running an aliquot of the preparation on a denaturing agarose gel and staining with ethidium bromide. RNA concentration was determined spectrophotometrically at 260 nm and 280 nm wavelengths. cDNA were transcribed from 1 μg of RNA with 2.5 μM Oligo dT and 200 U ExScript reverse transcriptase (TaKaRa, Japan, http://www.takara-bio.com) in a 20 μL reverse-transcription reaction mix containing 500 μM dNTP and 40 U RNase-Inhibitor (Sigma-Aldrich, St. Louis). RNA and Oligo dT were incubated for 5 min at 65°C in a thermal cycler and quickly chilled on ice. The thermo-profile of the cDNA generation was 42°C for 15 min and 95°C for 2 min, ending at 4°C.

#### Real-time PCR

The level of gene expression for nerve growth factor (*NGF*), brain-derived neurotrophic factor (*BDNF*) and neurotrophin-3 (NT-3) from NSC cultures at passages 5-7 were quantified using the ABI 7300 Real-Time PCR System (Applied Biosystems, Foster City, CA, http://www.appliedbiosystems.com) and the specific primer-pairs for BDNF (forward: 5'-ACC CTG AGT TCC ACC AGG TG-3', reverse: 5'-TGG GCG CAG CCT TCA T-3'), NGF (forward:5'-TGG ACC CAA GCT CAC CTCA-3', reverse: 5'-GGA TGA GCG CTT GCT CCT-3'), *NT-3 *(forward: 5'-GAT CTT ACA GGT GAA CAA GGT GAT G-3', reverse: 5'-TTG ATC CAT GTT GTT GCC TTG-3') and the house keeping gene *β-actin *(forward: 5'-CTA CAA TGA GCT GCG TGT GG-3', reverse:5'-CAG TCA GGA TCT TCA TGA GG-3'). The thermo-profile was 50°C for 2 min and 95°C for 10 min, followed by 40 cycles of 95°C for 15 s, 60°C for 1 min, and finally 95°C for 15 s, 60°C to 95°C for 30 s, and 95°C for 15 s. Quantification of the gene of interest was accomplished by measuring the threshold cycles and comparing them to the standard curve to determine the copy number. The process of calculating threshold cycles, preparing a standard curve, and determining the copy number was performed using system software. All measurements were performed in triplicate in two separate experiments.

#### Preparation of NSC for Transplant

NSC at passage 6 were labeled with 10 μM bromodeoxyuridine (Sigma-Aldrich, St. Louis) in the supplemented culture medium one day prior to transplantation to ischemic animals for *in vivo *study. BrdU-labeled cells were then trypsinized, washed and adjusted to 5 × 10^3^/μl in Dulbecco's phosphate-buffered saline (PBS; Boster, Hubei, China).

### Induction of focal cerebral Ischemia in rats

Animal treatments were designed to minimize pain or discomfort in accordance with the current protocols approved by the Chinese Medical Ethical Committee for animal welfare. Forty Sprague-Dawley rats (Weitonglihua, Beijing, China, http://www.vitalriver.com.cn) at a mean age of 14 weeks and body weight between 240 and 260 g were maintained on a 12-hour light/dark schedule and randomly assigned to the following four groups: (A) normal control with sham-operation (CG), (B) ischemic group with PBS injection (IG), (C) ischemic group treated with NGF (NGFG) and (D) ischemic group transplanted with NSC (NSCG). Animals were anesthetized with an intra-peritoneal injection of 400 mg/kg chloral hydrate (Pharmaceutical Plant of Tiantan Hospital, Beijing, China, http://www.bjtth.com). The rectal temperature was monitored and maintained at 37.5°C with a thermal pad throughout the surgical procedure. A scalp incision of 0.5 cm was made at one-third distal area between the left eye and ear. The temporalis was separated to expose the zygoma and squamosal bone. A burr hole of 1.5 × 2 mm was made using a 1 mm micro-drill rostal to the anterior junction of the zygoma and the squamosal bone. The dura mater was carefully pierced with an iris knife. The exposed middle cerebral artery was isolated and ligated using a 10-0 suture. After covering the burr hole with a piece of gelatin sponge, the temporalis and overlying skin were sutured. Animals were then placed in the supine position and a midline incision was made in the neck. The bilateral common carotid arteries were isolated. The left artery was ligated with 4-0 suture, whereas the right artery was occluded using a micro-aneurysm clip for 1.5 h. The skin was then sutured. Operated animals were kept individually for a day and then in a cage for six. All 30 lesioned rats showed signs of consciousness disturbance, including drowsiness, paucity of movement and coma. Sham-operated animals did not receive the ligation or occlusion.

### Transplantation

Seven days after the induction of focal cerebral ischemia, animals were anesthetized with an intra-peritoneal injection of 400 mg/kg chloral hydrate (Pharmaceutical Plant of Tiantan Hospital, Beijing, China). The rectal temperature was monitored and maintained at 37.5°C. A scalp incision was made behind the superior nuchal line at 0.5 cm. The posterior occipital muscle was separated to expose the atlanto-occipital membrane. 1.5 × 10^5 ^NSC at passage six were suspended in 30 μL PBS and injected into the cisterna magna through the atlanto-occipital membrane in NSCG. 30 μL of 10 ng NGF and PBS were injected into the cisterna magna in NGFG, and 30 μL PBS were injected into IG.

### Behavioral Assessment

#### Somatosensory response

Evoked potentials are the electrical signals generated by the nervous system in response to sensory stimuli. The measurement of somatosensory evoked potentials has been used in the diagnosis and prognosis of neurologic disorders[6]. Animals were anesthetized with chloral hydrate 5 days before and 2 days, 7 days, 14 days and 28 days after NSC transplantation or PBS injection. An incision of 0.8 cm was made in the midline of the skull. Bilateral burr holes were made at 1 mm posterior and 3 mm lateral to the bregma by using a micro-drill and 1 × 3 mm coordinate paper. Titanium alloy electrodes were placed into the burr holes and fixed with sterilized bone wax. The scalp was sutured. Somatosensory evoked potentials (SEPs) were measured using an Axon electrophysiology monitoring system (Axon Instrument, Sunnyvale, CA, http://www.axon.com). The stimulating electrode and the reference electrode were placed in the median nerve of the muscles between the ulner and radical bone, and the pre-frontal scalp of the midline, respectively. SEPs were obtained by electrical stimulation of 200 pulses at 2 Hz and 0.8 mA. The measured latency was the time span from the stimulation to the beginning of the first wave, whereas the amplitude was the voltage difference between the positive and the negative wave. The relative latency and amplitude are expressed as ratios of latencies and amplitudes of the intact side to the injured side, respectively.

#### Trap channel test

The trap channel test for the analysis of motor function in rats has been previously described elsewhere[7]. Briefly, animals were allowed to crawl three times along a horizontal channel made of Plexiglas that was 75 cm long, 10 cm wide and 10 cm high, with evenly placed 2-cm long stepping platforms. The motor functions of forelimbs and hind-limbs of rats with and without NSC transplantation or NGF or PBS injection were assessed on day 2, day 7, day 14 and day 28 by counting the number of foot faults in the left and right forelimbs. The sum of foot faults (SOFFF) in the forelimbs is defined as the number of faults of right and left forelimbs, whereas the differentiation of forelimb foot faults (DOFFF) is the difference between the number of faults for the right and left forelimbs. SOFFF and DOFFF were used as an index of motor deficit.

#### CSF aspiration

80 μL of CSF were aspirated from the cisterna magna of 40 chloral hydrate-sedated rats on week 4. The collected CSF was spun at 400 *g *for 10 min to remove cell contamination, and the supernatant was then stored at -80°C.

#### Tissue Processing for histology and immunohistochemical staining

Upon completion of the *in vivo *monitoring, rats were anesthetized with 600 mg/kg chloral hydrate. The thoraxes were cut open, and the animals were trans-cardiacally perfused with physiologic saline and 4% paraformaldehyde. The brains were then fixed in 30% sucrose. The freshly isolated brains were cut into 20 μm thick coronal slices with a cryo-mount and mounted onto poly-L-lysine-coated slides. To prepare the cells for immunohistochemical staining, cultures of NSC were enzymatically segregated with trypsin. After thorough washing, 1 × 10^5 ^separated cells suspended in PBS were cytospun on slides and kept at -80°C until staining.

### Immunohistochemistry staining

Immunohistochemical staining of nestin and BrdU were performed to assess the property of NSC in cultures and track the migration and homing of allogeneic BrdU-labeled NSC in the host brain. In addition to the expression of the class I and II MHC, the activation of caspase III in NSC cultures and brain sections was also assayed. Primary antibodies, including anti-nestin (1:150; Sigma-Aldrich, St. Louis), anti-BrdU (1: 400; Sigma-Aldrich, St. Louis), anti-class I MHC (1:200; AbD Serotec, NC, http://www.ab-direct.com), anti-class II MHC (anti-RT-1B 1:200; AbD Serotec, NC) and anti-caspase III (1:50, Abcam, Cambridge, UK, http://www.abcam.com), were employed. Incubation was conducted at 4°C for 24 hours. After extensive washing, signals were detected and visualized using HistostainTM-SP kit and the manufacturer's protocol (Zhongshan Beijing, China, http://gjj.cc/nongye/shengwugongcheng/zsbio.htm).

### Statistical Analysis

Results are expressed as mean ± standard deviation (SD). The non-parametric one-way ANOVA was applied to analyze continuous variables: the gene expression of *NGF*, *BDNF *and *NT-3 *derived from different culture passages, NGF concentration in different NSC culture passages and CSF aspirated from studied rats, relative latency and amplitude of SEP and the sum and differentiation of forelimb-foot fault of studied rats at different points. Data were analyzed using SPSS software version 11.5 (SPSS, IL, http://www.spss.com). Differences between groups were regarded as significant if *p *≤ 0.05.

## Results

### Primary NSC cultures

Primary cells segregated from the neural cortex of E16 Sprague-Dawley rat embryos formed free-floating neurospheres in the serum-free medium supplemented with FGF and EGF. Cultures were passaged after seven days. In two separate experiments of two replicate cultures at passages 1-7, the trypan blue dye exclusion tests revealed a cell viability of 79 ± 4.4% (range: 72 - 86%). Immunohistochemical staining demonstrated nestin-positive neurospheres up to passage 7, suggesting the successful *ex vivo *expansion of NSC (Data not shown).

### *In vitro *characterization of NSC

**Figure 1:**
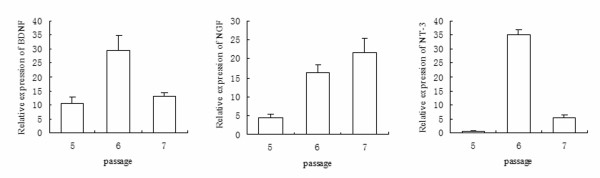
**Quantitative real-time PCR showing the relative expression of gene NGF (A), BDNF (B) and NT-3 (C) of triplicate culture passages five to seven of primary neural stem cells from cortex of E16 Sprague-Dawley rat embryos**.

Quantitative real-time PCR was conducted to determine the gene expression of neurotrophic factors in *in vitro *NSC cultures. Figure 1 shows the relative gene expression of *NGF*, *BDNF *and *NT-3 *in NSC culture at passages 5-7 in three separate experiments. A progressive increase of *NGF *was noted, and an up-surge of *BDNF *and *NT-3 *was seen in NSC culture at passage 6 (*p *< 0.0001).

**Figure 2:**
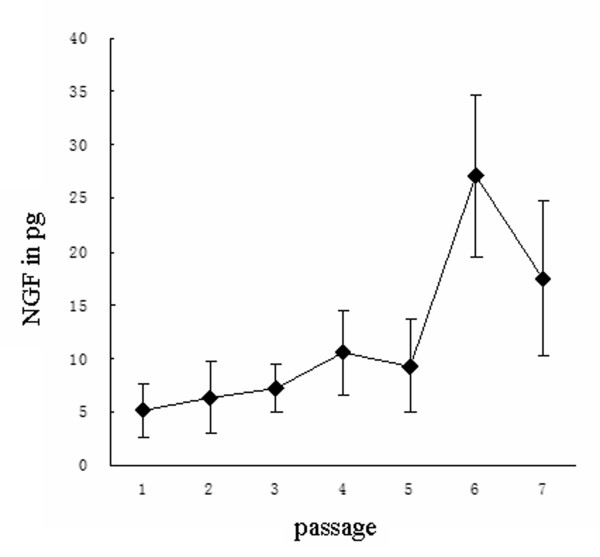
**ELISA of nerve growth factor in one-day spent tissue culture media of culture passages five to seven of primary neural stem cells from cortices of E16 Sprague-Dawley rat embryos in three separate experiments**. A preponderance of NGF in pg per day of culture of 1 × 106 cells was evident in culture passages six and seven.

ELISA was performed on one-day-old tissue culture medium from three replicate cultures at passages 1-7 in two separate experiments to evaluate the gene translation of NGF (Figure 2). A steady increase of NGF protein was noted from passages 1-5, followed by a peak of production at passage 6, which was significantly higher than those derived from earlier passages (*p *< 0.0001) and from subsequent cultures at passage 7 (*p *= 0.0006). Using NGF as a model, our data suggested an *in vitro *synthesis of neurotrophic factors in NSC cultures.

### *In vivo *Study of NSC-Derived Cells

#### Somatosensory function

NSC (1.5 × 10^5^) at passage 6 were transplanted into the cisterna magna of 10 ischemic rats seven days after the induction of focal cerebral ischemia. Transplanted animals did not behave abnormally or develop dyskinesia. Thirty ischemic rats with and without NSC transplant or NGF injection and 10 sham-operated rats were subjected to the electrophysiological tests before and after surgery. One ischemic control rat injected with PBS died after the first electrophysiological test. Compared to the 10 sham-operated normal control rats, SEP was not elicited among the 30 ischemic rats on day two after ischemic induction or day two after the NSC transplant or NGF administration. On day 7, a very weak SEP was observed among ischemic rats treated with either NSC or NGF but not in ischemic control rats (Table 1). A progressive increase of SEP in terms of the relative latency and amplitude was seen at week two and four in the ischemic control rats, suggesting regeneration. At weeks two and four, transplanted ischemic rats responded to the somatosensory stimuli more effectively than ischemic rats with NGF supplement and ischemic control rats, as shown by the relative latencies (*p *< 0.0001). The relative amplitudes derived from transplanted rats at week two were higher than those of the ischemic rats supplemented with NGF and the ischemic control rats (*p *= 0.0086), despite the fact that the relative amplitude of transplanted rats at week four was significantly lower than that of the sham-operated normal control (*p *< 0.0001). These data suggest that NSC transplant could improve the somatosensory response after ischemic stroke.

**Table 1:** Somatosensory evoked potential at different time points

	Relative Latency (Relative Amplitude) of Somatosensory Evoked Potential in Mean ± SD				
	
Period of NSC transplant/NGF injection	Day -5	Day 2	Week 1	Week 2	Week 4
Sham-operated normal rats (n = 10)	1.03 ± 0.02 (1.06 ± 0.33)	1.02 ± 0.02 (0.99 ± 0.32)	0.96 ± 0.04 (0.98 ± 0.24)	1.02 ± 0.04 (1.06 ± 0.29)	1.02 ± 0.04 (1.1 ± 0.40)
Ischemic control rats (n = 9)	-	-	-	1.64 ± 0.25 (0.36 ± 0.33)	1.42 ± 0.11 (0.58 ± 0.25)
Ischemic rats with NGF administration (n = 10)	-	-	1.83 ± 0.06 (0.22 ± 0.11)	1.52 ± 0.10 (0.34 ± 0.16)	1.24 ± 0.07 (0.64 ± 0.15)
Ischemia rats with NSC transplant (n = 10)	-	-	1.86 ± 0.14 (0.21 ± 0.13)	1.22 ± 0.09 (0.51 ± 0.21)	1.18 ± 0.04 (0.7 ± 0.17)

#### Motor function

**Figure 3:**
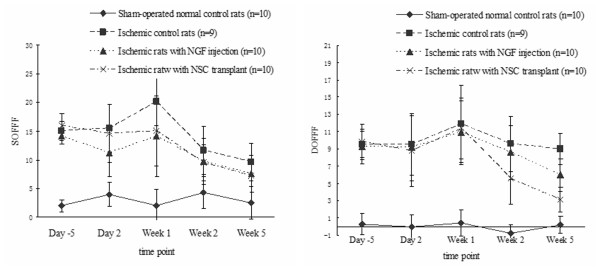
**Analyses of motor function over four weeks of sham-operated normal control rats, ischemic control rats and ischemic rats with either NGF injection or neural stem cell transplant**. A: sum of forelimb foot faults, B: differentiation of forelimb and foot faults.

Thirty ischemic rats with (n = 10) and without NSC transplant (n = 9) or NGF injection (n = 10), and 10 sham-operated rats were assessed over four weeks using a horizontal channel connected to a ladder. Forelimb faults were summed and differentiated. On examining the sum of forelimb and foot faults (SOFFF), the three groups of ischemic rats had higher scores than sham-operated normal control rats (Figure 3A). On day two, ischemic rats given the NGF injection showed the least motor impairment, as measured by the SOFFF, compared to their ischemic counterparts with or without NSC transplant (*p *= 0.025). On week one, the SOFFF was significantly lower in ischemic rats transplanted with NSC than ischemic control rats (*p *= 0.011), but was comparable to that of ischemic rats injected with NGF. The SOFFF of the three ischemic groups on week two and four were comparable to (*p *> 0.05) but higher than that of sham-operated normal control rats. Figure 3B shows the relatively stable, but significantly higher, coefficients of the differentiation of forelimb and foot faults (DOFFF) derived from ischemic control mice over four weeks compared to the sham-operated normal control rats. The DOFFF of the three groups of ischemic rats before week two were comparable (*p *> 0.05). From weeks two to four, the DOFFF of ischemic rats with NSC transplant was lower than that of rats injected with NGF, which was in turn lower than that of ischemic control rats (*p *< 0.05). This suggests that NSC transplant and NGF administration could enhance symptomatic relief.

#### NGF synthesis

ELISA of NGF in the CSF aspirated from the IG on day 28 after sham operation displayed a physiological level of 0.44 ± 0.38 pg/mL. The NGF in CSF aspirated on the same time line from 9 ischemic control rats and 10 ischemic rats injected with NGF were 14.25 ± 5.21 pg/mL (32.4-fold increase) and 16.22 ± 4.43 pg/mL (36.9-fold increase), respectively. The NGF in the CSF at week four of 10 ischemic rats given NSC transplant was 37.86 ± 4.12 pg/mL, which was 2.7-fold and 86-fold higher than those derived from untreated ischemic rats and control rats, respectively. These data suggest an ischemia-mediated up-regulation of *in vivo *NGF synthesis that is augmented by the NSC allograft.

#### Histology

**Figure 4:**
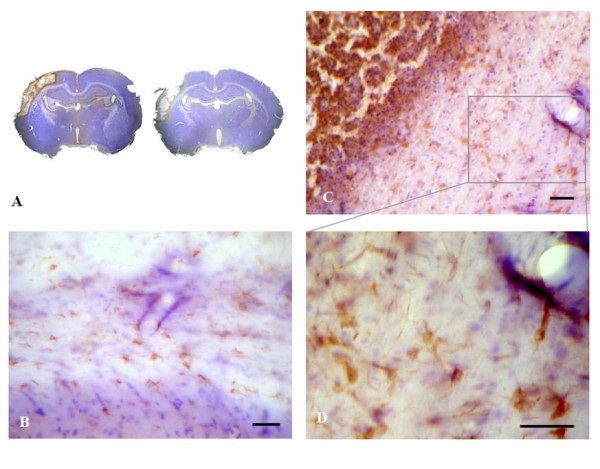
**Tracking of BrdU-labeled neural stem cells at passage six in the ischemic brain of rat having undergone cell therapy for four weeks**. A: a representative coronal section of a transplanted rat demonstrated the localization of reddish-brown colored BrdU^+ ^cells (left panel) to the cortex, hippocampus, striatum and brain parenchyma near the third ventricle, and of a sham-operated normal control rat without BrdU postivity (right panel) B: migration of BrdU^+ ^cells along the corpus callosum. C and D: small clusters of BrdU^+ ^cells displaying a glial morphology. Scale bar: 75 μm.

Animals were sacrificed on week four after CSF aspiration and the completion of behavioral assessments. Tracking of BrdU^+ ^NSC revealed that a majority of the donor cells engrafted to the infarcted areas of the cortex, hippocampus, striatum and parenchyma near the third ventricle (Figure 4). Migration of the BrdU^+ ^cells along the corpus callosum and the ventricular wall was noted. Small clusters of BrdU^+ ^cells and BrdU^+ ^cells with glial morphologies of 10-20 μm in size were also evident.

**Figure 5:**
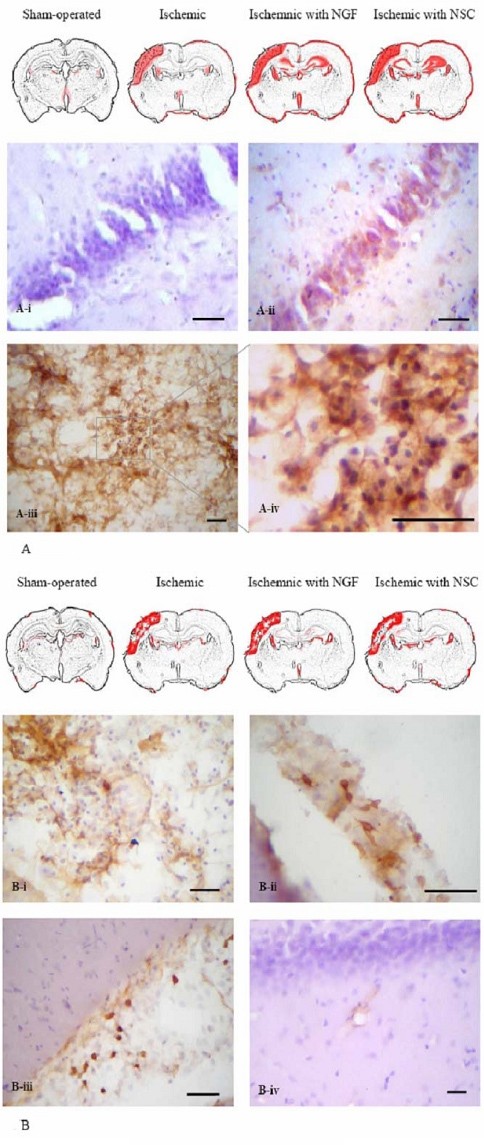
**Immunohistochemical staining of MHC**. Reddish-brown immunoreactivity of class I and II MHC were shown in panel A and B, respectively. A representative coronal section of the hippocampus of an ischemic rat brain without injection of NGF or NSC exhibited no positivity of class I MHC (A-i). Intense staining of class I MHC was noted in the cytoplasm of pyramidal neurons in the hippocampus of ischemic rats undergone neural stem cell transplant for four weeks (A-ii). Clusters of cells with class I MHC-positivity were evident in the infarcted brain parenchyma of transplanted rats (A-iii and A-iv). A comparable extent of class II MHC was noted in ischemic rats irrespective of any therapy but unremarkable in normal rat (panel B, top row). Reddish-brown staining of Class II MHC was evident in the infarcted brain parenchyma (B-i), along the meninge (B-ii), areas near the ventricular lining and vascular wall near the hippocampus (B-iv) of transplanted rats. Scale bars: 75 μm

Immunohistochemical staining of class I MHC demonstrated high expression levels in the lesioned cortex and brain parenchymas near the ventricular lining in the three groups of ischemic rats, which was in marked contrast to the low expression in normal rats (Figure 5A). In addition, the class I MHC was detected in the hippocampus of ischemic rats with either NGF injection or NSC transplant, but not in control brains or ischemic brains without therapy. The intensity was more profound in the NSC-transplanted group than in the NGF-injected group. Figure 5B shows that a small amount of the class II MHC was detected in normal brain tissue, but was up-regulated under ischemic stress. The extents of the class II MHC immunoreactivity were comparable among the three groups of ischemic rats, irrespective of the treatments given. These data suggest that ischemia might up-regulate MHC expression, and that the class I MHC may be further uplifted by NGF supplement or NSC transplant.

**Figure 6:**
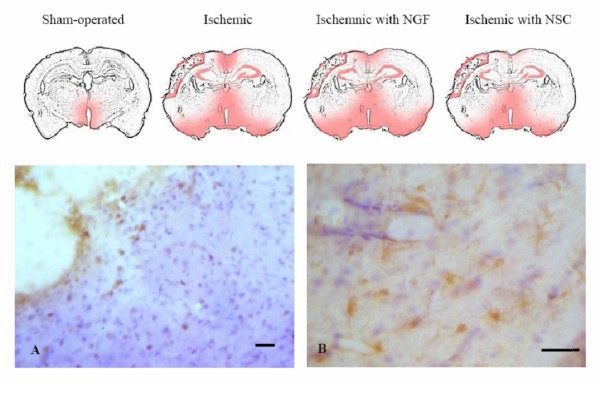
**Immunohistochemical staining of caspase III**. Weak reddish-brown immunoreactivity was demonstrated in neural tissues near the third ventricle of sham-operated normal control rats, whereas strong reactivities were evident in the cortex, hippocampuses, striata and neural tissues close to lateral ventricles and third ventricles of ischemic rats with and without NGF administration or neural stem cell transplant. A: reddish brown coloration of caspase III immunoreactivity along the ventricular lining of cells of ischemic rat having undergone neural stem cell transplant for four weeks, B: caspase III+ cells took the glial morphology. Scale bar: 75 μm.

Immunoreactivity of caspase III was almost non-existent in the control brain parenchyma, except in the neural tissue adjacent to the third ventricle. Conversely, a high level of caspase III was noted in the cortex, hippocampus, striatum and neural tissue around the third ventricle of ischemic rats with and without either NGF administration or NSC transplant (Figure 6). The extent of caspase III immunoreactivity was comparable among the three groups of ischemic rats.

## Discussion

In this study, we found an up-regulated expression of the class I and II MHC in rat brains under ischemic stress. The extent of the class I MHC augmentation was more remarkable in ischemic rats given an NSC transplant than in rats given an NGF supplement, whereas class II MHC expression was comparable among ischemic rats irrespective of NGF or NSC therapy. *In vitro *and *in vivo *analyses of NSC-derived NGF demonstrated that the NSC-derived, neurotrophin-modulated MHC expression correlated with the degree of transient symptomatic relief in stroke rats and promoted no secondary injury, such as apoptotic cell death and inflammation [8,9].

Data from our present and previous studies demonstrate that a minority of implanted donor stem cells can migrate along nerve fiber bundles, home to lesioned brain parenchymas and differentiate into mature cells of interest [3,10,11]. The low degree of differentiation and integration of the transplanted cells in the parenchyma often correlated poorly with the improved functional benefits [12,13]. As there is little evidence of neuronal replacement, other mechanisms might account for the functional recovery. Neurotrophin genes have been reported to be expressed and transcribed by NSC *in vitro *[14]. The administration of neurotrophin-secreting stem cells or neurotrophic factors might be a potential alternative [15,16].

Neurotrophins, including NGF, BDGF, NT-3, NT-4 and others, are a group of short-lived proteins in the CNS, which are key regulators of cell fate and cell shape [17,18]. The growth-enhancing effects of neurotrophins have also been reported [19]. In this study, we provide evidence both *in vitro *and *in vivo *of neurotrophin production by NSC and confirmed the constitutive secretion that was proposed by Lu *et al*. [20]. Interestingly, we noted an increase of NGF in the CSF of rats after ischemic stress. The extent was further amplified in ischemic rats that were given a NSC transplant. The high dose of NGF might have a neuroprotective effect on the injured brain to prevent further secondary injuries, as suggested in this study and that of Chiaretti *et al*. [21]. The up-regulation of the class I MHC correlated well with the symptomatic relief in ischemic rats given the NSC transplant and the upsurge of NGF *in vivo*, suggesting an immuno-modulation of the class I MHC by NSC-derived neurotrophins in the micro-environment of the lesioned brain parenchyma.

The MHC is a family of molecules that are responsible for the immune recognition and are particularly important in the context of the adaptive immune response. The anergy of the regulatory MHC when presenting inflammatory elements to immuno-competent cells in the CNS might do more harm than good. Mounting evidence suggests that some forms of immunologic intervention can help protect or restore CNS integrity [22]. The present study shows that an NSC allograft might boost neural regeneration during focal cerebral ischemia in a rat model via the immuno-modulation of class I MHC expression by NSC-mediated neurotrophins and eventually lead to functional recovery without activating the caspase III inflammatory response. Recently, the class I MHC was found to be crucial to neural development, neuronal differentiation, synaptic plasticity and behavior [23]. Thus, manipulating and targeting MHC signaling might facilitate NSC-derived neurotrophin-mediated functional restoration after stroke. This possibility should be elucidated and explored in future studies.

## Conclusions

The findings presented here provide further insights into the mechanisms of NSC in the regeneration of the CNS. Should the MHC modulation mediated by NSC-derived neurotrophins be elucidated, strategic cellular therapy for neural injuries and neuro-degenerative diseases may be revolutionized, and novel treatment modalities could be developed.

This paper is not based on a previous communication to a society or meeting.

## Competing interests

The authors declare that they have no competing interests.

## Authors' contributions

CRS conceived of the study, participated in some parts of the research and wrote the manuscript. HZ carried out the histology test and participated in ELISA test. JL participated in the in vitro characterization of NSCH. Huang carried out the culture of NSC and participated in the motor function test. HBC participated in the creating the animal models. YJW participated in ELISA test. PL carried out the electrophysiology test. YHA participated in its design and coordination and helped to draft the manuscript. All authors read and approved the final manuscript.
